# Increased plasminogen binding is associated with metastatic breast cancer cells: differential expression of plasminogen binding proteins.

**DOI:** 10.1038/bjc.1998.261

**Published:** 1998-05

**Authors:** M. Ranson, N. M. Andronicos, M. J. O'Mullane, M. S. Baker

**Affiliations:** Department of Biological Sciences, University of Wollongong, NSW Australia.

## Abstract

**Images:**


British Journal of Cancer (1998) 77(10), 1586–1597
© 1998 Cancer Research Campaign

### Increased plasminogen binding is associated with metastatic breast cancer cells: differential expression of plasminogen binding proteins

**M Ranson, NM Andronicos, MJ O'Mullane and MS Baker**

Department of Biological Sciences, University of Wollongong, Wollongong, NSW Australia 2522

**Summary** Overexpression of urokinase-type plasminogen activator and its receptor correlates with metastatic capacity in breast cancer. In this study we show that the urokinase/urokinase receptor-overexpressing, metastatic human breast cancer cell line MDA-MB-231 (1) bound significantly more cell-surface plasminogen in a lysine-dependent manner and (2) was capable of generating large amounts of plasmin compared with the non-metastatic cell lines MCF-7 and T-47D. In addition, distinct plasminogen binding proteins were detected in the plasma membranes of the cell lines, suggesting heterogeneity of binding proteins. Plasminogen binding was analysed using a combination of dual-colour fluorescence flow cytometry and ligand histochemistry (for comparative and cellular localization of ligand binding), and fluorimetry (for Scatchard analysis). Apart from revealing the greater plasminogen binding capacity of MDA-MB-231 cells, flow cytometry and histochemistry also revealed that, in all three cell lines, non-viable or permeabilized cells bound significantly more plasminogen in a lysine-dependent manner than viable or non-permeabilized cells. Viable MDA-MB-231 cells bound plasminogen with moderate affinity and high capacity ($K_d$ = 1.8 μM, receptor sites per cell 5.0 × $10^7$. Our results indicate that differences in cell surface-specific plasminogen binding capacity between cell lines may not be detectable with binding techniques that cannot distinguish between viable and non-viable cells.

**Keywords:** plasminogen receptor; plasminogen activation; breast cancer cell; plasminogen binding protein

The acquisition of the malignant phenotype requires a multitude of complex processes, including a loss of control of cell proliferation (e.g. via oncogene activation), higher metabolic requirements and the ability of cells to invade and metastasize throughout the body (reviewed in Evans, 1991). The processes of tumour cell invasion and metastasis are likely to involve the inappropriate expression of proteinases (reviewed in Mignatti and Rifkin, 1993). The serine proteinases of the plasminogen activation proteolytic cascade appear to play an important role in this process (reviewed in Duffy, 1993; Mignatti and Rifkin, 1993). Plasminogen, a broad-spectrum serine endopeptidase zymogen, is secreted in its mature form as a single-chain glycoprotein containing a N-terminal glutamic acid, a binding domain [consisting of five kringle structures on which the lysine binding sites (kringles 1, 4 and 5) are located] and a protease domain (reviewed in Castellino, 1995). When activated by urokinase plasminogen activator (uPA) or tissue plasminogen activator, plasminogen is converted into the active twin-chain proteinase, i.e. plasmin (Castellino, 1995). It is clear that tissue plasminogen activator and plasminogen bind to and mediate fibrin clot dissolution, whereas uPA is primarily involved in pericellular proteolysis as it binds to specific cell-surface glycosyl phosphatidylinositol-anchored receptors (uPAR) (Moller, 1993).

The uPA–uPAR interaction on cancer cell surfaces is a key event that results in increased in vitro matrix degradation and migration (Duffy, 1993; Mignatti and Rifkin, 1993). In the absence of extracellular proteolysis, inactive uPA binding has been shown to induce cell migration by signal transduction events via uPAR (Busso et al, 1994). Nevertheless, dissolution of matrix via plasminogen activation at the cell surface is important for metastasis to occur. Indeed, the inhibition of uPA and plasmin by specific inhibitors or antibodies, or antisense inhibition of uPAR resulted in decreased plasminogen activation and hence decreased extracellular matrix degradation in vitro or decreased metastasis in nude mouse models with various cancer cell lines (Baker et al, 1990; Kook et al, 1994), including human breast cancer cell lines (Holst-Hansen et al, 1996; Stonelake et al, 1997). In vitro invasiveness has been correlated with uPA and plasmin activity as well as high uPAR and plasminogen activator inhibitor type 1 (PAI-1) protein levels in human breast cancer cell lines (Holst-Hansen et al, 1996). Long-term studies have shown that increased primary breast cancer biopsy levels of the uPA antigen are related to shorter disease-free period and reduced survival in women with breast cancer (Duffy, 1993). It is now apparent that the combined overexpression of uPA, uPAR and plasminogen activator inhibitor type 1 (PAI-1) in human breast carcinoma tissue is associated with breast cancer progression and can also be related to shorter disease-free period and reduced survival (Janicke et al, 1991; Christensen et al, 1996; Costantini et al, 1996; Nielsen et al, 1996), while elevated PAI-2 levels are related to a favourable prognosis in primary breast cancer (Foekens et al, 1995).

Plasminogen has been immunologically localized to cell surfaces in sections of human mammary carcinoma tissue (Burtin et al, 1993) and to the invasive front of cutaneous melanoma lesions (De Vries et al, 1996). Cell-surface localization of plasminogen would be advantageous for cell migration, as the activation of receptor-bound plasminogen to plasmin is enhanced while cell-bound plasmin is protected from circulating inhibitors (e.g. $\alpha_2$-antiplasmin) (Plow and Miles, 1990). Plasminogen binds to a

*Received 9 April 1997*
*Revised 15 July 1997*
*Accepted 2 September 1997*

*Correspondence to:* M Ranson number of different cell types via its kringle domains within a range of affinities (dissociation constants ($K_d$) = 0.1–5 μM) and receptor sites per cell ($10^4$–$10^8$), and various candidate receptors have been identified from endothelial cells (Cesarman et al, 1994), neurons (Parkkinen and Rauvala, 1991), monocytoid cells (Miles et al, 1991) and breast cancer cells (Hembrough et al, 1995). Plasminogen also interacts with several extracellular matrix components, such as type IV collagen (Stack et al, 1992), and with non-proteinaceous cellular moieties, such as gangliosides (Miles et al, 1989).

Approximately 20–30% of all human breast cancers overexpress two closely related receptor tyrosine kinases, epidermal growth factor receptor (EGFR) and/or p185$^{HER2/neu}$ (c-*erb*B-2), and this phenotype is associated with more aggressive tumour growth and reduced patient survival (Singleton and Strickler, 1992). In contrast, breast cancer cell lines that are oestrogen receptor (ER) positive and have little or no EGFR have a fundamentally non-metastatic phenotype (Lee et al, 1990). The presence of ER is also inversely related to uPA and uPAR levels in breast carcinomas and cell lines (Mignatti and Rifkin, 1993; Long and Rose, 1996). In addition, it is apparent that breast cancer cell lines that readily form tumours and metastasize in nude mouse models and/or are invasive in in vitro models of metastases (e.g. MDA-MB-231 cells) (Thompson et al, 1992) tend to be EGFR(+)/*erb*B-2 protein(+) and ER(–) (Lee et al, 1990), as well as uPA/uPAR(+) (Holst-Hansen et al, 1996). Interestingly, EGFR stimulation or c-*erb*B-2 overexpression in human breast cancer cells enhances the expression and secretion of uPA and expression of uPAR (Long and Rose, 1996; Connolly and Rose, 1997).

The purpose of this study was to characterize the cell-surface plasminogen binding events in three human breast cancer cell lines. We report that in these cell lines, elevated cell-surface lysine-dependent plasminogen binding and plasmin formation is associated with metastatic capacity and other parameters (EGFR/*erb*B-2 status, ER status, uPA/uPAR status) commonly associated with human breast cancer malignancy. We also found that non-viable cells bound 100-fold more plasminogen than viable cells and that the presence of non-viable cells must be considered when determining cell-surface binding parameters. In addition, a distinct plasminogen receptor profile was evident in the plasma membranes of the breast cancer cell lines, suggesting heterogeneity of plasminogen binding proteins in these cell lines.

### MATERIALS AND METHODS

### Materials

RPMI 1640, L-glutamine and Hank's buffered salt solution were purchased from Trace Biosciences (Castle Hill, NSW, Australia). Fetal calf serum was obtained from CSL (Parkville, Victoria, Australia). Tranexamic acid, ε-amino-n-caproic acid (EACA), aprotinin, polyvinyl pyrrolidine (PVP-40), bovine serum albumin (fraction V) (BSA) and fluorescein isothiocyanate (isomer 1) (FITC) were purchased from Sigma Chemical (St Louis, MO, USA). Biotin-X-NHS was from Calbiochem (San Diego, CA, USA). Z-lysine thiobenzylester was from Peninsula Laboratories (CA, USA). Enhanced chemiluminescence (ECL) detection kit was purchased from Amersham International (Buckinghamshire, UK). Molecular weight protein standards were obtained from Novex (San Diego, CA, USA). The Dako LSAB+ kit was from Dako (CA, USA).

### Specific proteins and antibodies

Human recombinant α-enolase was prepared as previously described (Andronicos et al, 1997). Active human uPA was from Serono (Sydney, NSW, Australia). Plasminogen, plasmin, the plasmin-specific substrate Spectrozyme-PL, mouse anti-human uPAR monoclonal antibody (no. 3696) and rabbit anti-human uPAR polyclonal antibody (no. 399R) were purchased from American Diagnostica (Greenwich, CT, USA). Rabbit anti-human plasminogen polyclonal antibody and horseradish peroxidase-conjugated goat anti-rabbit polyclonal antibody were purchased from Calbiochem (San Diego, CA, USA). Mouse anti-human EGFR monoclonal antibody (clone LA1) was obtained from Upstate Biotechnology (Lake Placid, NY, USA), while mouse anti-human *Neu* (9G6) monoclonal antibody was obtained from Santa Cruz Biotechnology (Santa Cruz, CA, USA). SBU-LCA $IgG_{2a}$ monoclonal antibody against sheep lymphocyte markers was from the Centre for Animal Biotechnology (Melbourne, Victoria, Australia), and FITC-conjugated anti-mouse IgG was from Silenus (Sydney, NSW, Australia).

### Cell culture

The human breast cancer cell lines used in this study (MCF-7, MDA-MB-231, T-47D) were gifts from Professor R Sutherland (Garvan Institute of Medical Research, Sydney, NSW, Australia). The cell lines were all routinely cultured in RPMI-1640 supplemented with 10% (v/v) heat-inactivated fetal calf serum. The T-47D cell line was cultured in the above media containing insulin (0.2 IU $ml^{-1}$). The cells were incubated in a humidified incubator at 37°C with a 5% carbon dioxide–95% air atmosphere.

### Whole-cell lysate preparation

Confluent cells were washed with sterile ice-cold phosphate-buffered saline (PBS) and harvested by scraping with a rubber policeman. The cells were incubated with cell lysis buffer [50 mM Tris-HCl (pH 8.0), 150 mM sodium chloride, 4 mM magnesium chloride, 10% glycerol, 5 mM EDTA, 1 mM phenylmethylsulfonyl fluoride, 0.1% (v/v) Triton X-100, 0.5% (w/v) sodium deoxycholate] at 4°C for 20 min. The resulting lysates were collected and centrifuged at 14 000 ***g*** for 15 min at 4°C and the supernatant stored at –20°C until required.

### Membrane preparations

#### *Total cellular membrane*

Confluent cells were washed and harvested by scraping in ice-cold PBS and subjected to hypotonic shock in a small volume of ice-cold hypotonic buffer [3 mM sodium phosphate (pH 7.4), 5 mM EDTA, 1 mM phenylmethylsulfonyl fluoride]. The suspensions were then sonicated with several 20-s bursts at high power with a Branson Sonifier 250 (CT, USA), checked under a microscope for effective disruption of the cells and then centrifuged at 500 ***g*** for 5 min. The supernatant was centrifuged at 46 000 ***g*** for 30 min and the resultant supernatant was frozen in aliquots at –70°C. The crude membrane pellet was resuspended in 5 ml of ice-cold 5 mM EDTA/PBS, briefly resonicated, diluted to 50 ml with EDTA/PBS and centrifuged at 46 000 ***g*** for 30 min. The resulting membrane pellet was resuspended in 1 ml of EDTA/PBS with brief sonication, then aliquoted and stored at –70°C.

© Cancer Research Campaign 1998 

*Plasma membrane*

Confluent cells were washed, harvested, subjected to hypotonic shock as above and then homogenized in a Dounce homogenizer. Plasma membranes were then isolated using the aqueous two-phase PEG polymer/dextran system as described by Rana and Majumder (1987).

### Western blotting and plasminogen ligand blotting

Whole-cell lysates and membrane preparations were boiled in sample buffer, fractionated on 10% or 12% SDS-PAGE gels, and parallel gels were either stained with Coomassie blue or transferred to PVDF membranes at 100 V for 1 h or 30 V overnight at 4°C. For Western blotting, the membranes were washed in TBST [50 mM Tris-HCl (pH 8.0), 150 mM sodium chloride, 0.05% (v/v) Tween-20], blocked in 10% milk powder/TBST at room temperature for 1–2 h, rinsed in TBST, then incubated with the primary antibody in 2% milk/TBST for 1 h at room temperature. After extensive washing, the membrane was re-blocked for 20 min with 6% milk/TBST and incubated with the appropriate secondary antibody diluted 1:2000 in 2% milk/TBST. After three washes with TBST and one wash with TBS, the immune complexes were detected by ECL.

For ligand blotting, membranes were washed once with TNCM [50 mM Tris-HCl (pH 7.5), 150 mM sodium chloride, 2 mM calcium chloride, 3 mM magnesium chloride] and blocked with TNCM/2% PVP-40 overnight at room temperature. The membranes were then probed with 5 nM glu-plasminogen in the absence or presence of 100 mM EACA in TNCM/PVP-40 containing 0.05% (v/v) Tween 20 (TNCMT) for 45 min and washed for 1 h with three changes of TNCMT. After re-blocking for 30 min, a 1:2000 dilution of rabbit antiplasminogen polyclonal antibody in TNCMT/PVP-40 was added and incubated with the membrane for 1 h. This was followed by three 5-min washes, re-blocking for 10 min and probing with a 1:1000 dilution of horseradish peroxidase-conjugated goat anti-rabbit polyclonal antibody in TNCMT/PVP-40 for 1 h. This was followed by three washes with TNCMT and one wash with TNCM. The blots were then developed by ECL.

### Urokinase plasminogen activator activity and plasmin activity assays

The activity of uPA in the membrane preparations was measured as described by Coleman and Green (1981). Briefly, membrane preparations (10 μg of protein per well) were preincubated with glu-plasminogen (1 μM) for 45 min at 37°C, followed by a further 45-min incubation with the chromogenic plasmin substrate, Z-lysine thiobenzylester (170 μM). Colour development was read at 414 nm. Standard curves were constructed using uPA. Plasmin activity was measured using the Spectrozyme-PL assay (American Diagnostica; conditions are described in the figure legend). Standard curves for the uPA and plasmin assays were constructed using uPA and plasmin respectively.

### Flow cytometry and fluorimetry

Human glu-plasminogen, aprotinin or BSA in PBS were conjugated with FITC as described by Goding (1976). Unconjugated FITC was separated by a PD-10 gel filtration column equilibrated with PBS/0.1% azide. FITC-glu-plasminogen was able to bind lysine-Sepharose 4B, while FITC-aprotinin was able to inhibit plasmin activity, indicating that FITC conjugation did not inhibit the function of these proteins.

Subconfluent, adherent cells that had been in culture for 48 h without a change of media were harvested by rinsing flasks twice with cold PBS (pH 7.2) and then detaching with 5 mM EDTA/PBS at 37°C for 5 min. For FITC-glu-plasminogen binding assays, cells were washed twice and resuspended in freshly made and chilled binding buffer (Hanks buffered salt solution containing 1 mM

**Table 1** Metastatic phenotype of three human breast cancer cell lines

| Cell characteristic | Method of analyses[b] | Cell lines[a] | | |
|---|---|---|---|---|
| | | MDA-MB-231 | MCF-7 | T-47D |
| Morphology | Refer to Figure 3 | Spindle-shaped | Epithelial | Epithelial |
| Invasion and metastases in nude mice | Local invasion and lymph node involvement and/or presence of distant metastases | Yes[c] (3/5 mice) | No (0/8 mice) (0/20)[c,d] | No[c,d] |
| EGFR | Western blot[e,f] | ++++ | + | – |
| *neu*/c-*erb*B-2 | Western blot[e,g] | ++ | + | – |
| Oestrogen receptor | Immunohisto chemistry[h] | – | +++ | ++ |
| uPAR | Western blot[e,i]<br>Flow cytometry[j] | ++++<br>63 ± 7 MFI | ++<br>10 ± 3 MFI | +<br>4 ± 0.3 MFI |
| uPA activity (mIU $mg^{-1}$) | Coleman and Green assay[k] | 273 ± 14 | 59 ± 8 | 14 ± 0.4 |

[a]All values shown are taken from representative experiments. [b]Refer to Materials and methods for detail. [c]Tumours were often vascularized and lymph nodes enlarged. [d]Information obtained from Thompson et al (1992). [e]Band intensity was scanned and quantified by densitometry (Molecular Analyst Software, Bio-Rad) with blanks subtracted from all values. The intensities were rated as: –, negative; +, weakly positive; ++, moderately positive; +++, strongly positive; ++++, very strongly positive. [f]A major band of approximately 170 kDa detected. Performed with total and/or plasma membrane preparations. [g]A single major band of approximately 185 kDa detected. Performed with total and/or plasma membrane preparations. [h]Intensity of brown staining rated as: –, negative; +, weakly positive; ++, moderately positive; +++, strongly positive; ++++, very strongly positive. [i]A major band of approximately 55 kDa detected. Performed with total and/or plasma membrane preparations. [j]MFI, mean fluorescence intensity. Values shown are means ± s.d. ($n \geq 3$). [k]Performed with total membrane preparations. Values shown are means ± s.d. ($n \geq 3$).

 © Cancer Research Campaign 1998

**Figure 1** Cell-surface lysine-dependent plasminogen binding capacity of breast cancer cell lines. Cells were incubated with FITC-glu-plasminogen (0.5 μM) in the absence (total binding) or presence (lysine-independent binding) of 1 mM tranexamic acid, washed, resuspended in buffer containing propidium iodide and analysed by dual-colour flow cytometry. (**A**) The bar graph shows lysine-dependent binding (obtained by subtracting lysine-independent binding from the total binding) calculated from individual histogram plots in which the fluorescence intensity values were gated to include viable cells only. Values shown are means ± s.d. ($n$ = 4). (**B**) A representative density plot of fluorescence intensities due to FITC-glu-plasminogen binding (FL1-FITC) vs propidium iodide uptake (FL2-PI) in MDA-MB-231 cells. Viable and non-viable cell gates were set around cells that excluded and included PI respectively. (**C**) Representative histogram plots of FITC-glu-plasminogen binding to viable MDA-MB-231 cells. Plg, FITC-glu plasminogen; Plg + TA, Plg and tranexamic acid. No shift in fluorescence intensities relative to autofluorescence was seen when cells were incubated with 0.5 μM FITC-BSA

calcium chloride, 1 mM magnesium chloride, 20 mM HEPES (pH 7.4) and 0.1% BSA) at a concentration of $1 \times 10^6$ cells $ml^{-1}$. A 200-μl aliquot of the cell suspension was pelleted and resuspended in 200 μl of binding buffer containing FITC-glu-plasminogen in the presence or absence of the lysine analogue tranexamic acid. After incubation for 1 h on ice in the dark, the cells were washed and resuspended in 250 μl of binding buffer containing the non-vital dye propidium iodide (5 μg $ml^{-1}$). Cell-associated fluorescence was then measured by dual-colour flow cytometry (FACSort, Becton-Dickinson). By using dual-colour flow cytometry it was possible to distinguish between two parameters based on the different fluorochromes, i.e. ligand binding (FITC produces a strong green fluorescence) and cell viability (propidium iodide binds to DNA and dsRNA and produces a strong red colour) (Darzynkiewicz et al, 1994). This technique was used to establish 'gates' – the exclusion of propidium iodide for a viable 'gate', the inclusion of propidium iodide for a non-viable 'gate'.

Flow cytometry is a semi-quantitative technique and ligand binding, measured in fluorescence units, cannot be related to input protein concentration. Therefore, in order to analyse specific binding isotherms by Scatchard transformation, the binding experiments described above were analysed by fluorimetry. Briefly, after the final wash step, the cells were resuspended in 2 ml of binding buffer without propidium iodide, and the extrinsic fluorescence of FITC-glu-plasminogen was measured using a fluorimeter (F-4500, Hitachi) with a slit width of 0.5 mm. Excitation and emission wavelengths were set at 488 nm and 521 nm respectively. A FITC-glu-plasminogen standard curve was constructed to relate fluorescence units to plasminogen concentration taking into account the ratio of FITC per molecule of plasminogen. To determine the proportion of non-viable cells, parallel dual-colour flow cytometry experiments were performed and they consistently indicated that 10–15% of the cell samples were non-viable according to propidium iodide uptake.

Cell-surface plasmin was detected using dual-colour flow cytometry as described above with the following modifications. Cells were preincubated in the absence or presence of unlabelled glu-plasminogen (0.5 μM) for 30 min at room temperature followed by incubation on ice for 1 h with FITC-aprotinin (1 μM) in the absence or presence of a 50-fold excess of unlabelled aprotinin. The cells were then washed, resuspended in buffer containing propidium iodide and measured as described above.

For the detection of cell-surface uPAR, indirect immunofluorescence staining was performed. Cells were incubated with either an irrelevant isotype control (SBU-LCA IgG2a) or anti-human uPAR monoclonal antibody for 30 min on ice (10 μg $ml^{-1}$ in cold RPMI/0.1% BSA), washed with 1 ml of cold RPMI/0.1% BSA and incubated with FITC-conjugated anti-mouse IgG (1:50 dilution of stock in cold RPMI/0.1% BSA) for 30 min on ice in the dark. The cells were washed again, resuspended in 0.5 ml of PBS/0.1% sodium azide containing 5 μg $ml^{-1}$ propidium iodide, and the cells were immediately analysed by dual-colour flow cytometry as described above.

In all the fluorescence-based experiments, autofluorescence was subtracted. All data was analysed using CELLQuest software (Becton-Dickinson).

### Plasminogen ligand histochemistry

Cells were passaged onto sterile glass coverslips in their appropriate media and allowed to adhere and spread for at least 48 h.

© Cancer Research Campaign 1998

The cells were then washed three times with PBS and fixed with glutaraldehyde (1% (v/v) in PBS) for 1 h at room temperature. Fixation was necessary as unfixed cells consistently rounded up and floated off the coverslips. After washing twice with PBS, the cells were either permeabilized by incubation with 0.2% (v/v) Triton X-100 for 2 min at room temperature (to allow ligand to reach intracellular binding moieties in the glutaraldehyde-fixed cells; Harlow and Lane, 1988) or left in PBS. After two washes with PBS, the permeabilized and non-permeabilized cells were incubated with 3% hydrogen peroxide for 5 min, rinsed with PBS and incubated in PBS/0.1% BSA containing either 0.2 μM biotinylated BSA or plasminogen (prepared as previously described by Andronicos et al, 1997) in the absence or presence of 5 mM tranexamic acid for 30 min at room temperature. The cells were washed three times with PBS, incubated with streptavidin peroxidase and finally with the diaminobenzidine chromogen substrate solution provided with the Dako LSAB+ kit. After rinsing with PBS, the cells were viewed using a video camera (National Panasonic) attached to an inverted compound microscope (Leica, Germany). Colour images of the cells (original magnification ×400) were captured by a Power PC (Macintosh 8500/20) using Apple Video Player software (Macintosh).

### Invasive and metastatic characteristics of human breast cancer cell lines

Local tissue invasion and metastatic ability of the MDA-MB-231, MCF-7 and T-47D cell lines in nude mice has been characterized in detail by Thompson et al (1992). Only the MDA-MB-231 cell line was found to be invasive and metastatic. To confirm the in vivo characteristics of these cell lines 1–2 × $10^6$ cells per site were injected into the neck region (s.c.) of male 4- to 6-week-old Swiss *nu/nu* nude mice. Animals were sacrificed when tumours became 2.5 cm in diameter or after 60 days, whichever arose first. Primary tumours and adjacent lymph nodes were excised and examined histologically.

### RESULTS

### Metastatic phenotypes of human breast cancer cell lines

In order to confirm that the metastatic phenotypes of the human breast cancer cell lines MDA-MB-231, MCF-7 and T-47D concurred with published data, the invasive and metastatic ability of these cell lines in a nude mouse model were compared with morphology, EGFR, c-*erb*B-2/*neu*, ER and uPAR expression, and uPA activity (Table 1). These particular characteristics of the three breast cancer cell lines have not been compiled previously in one study but each characteristic was highly comparable to published data from several sources (Lee et al, 1990; Thompson et al, 1992; Holst-Hansen et al, 1996). The MDA-MB-231 cells had a spindle-shape morphology, were EGFR/*neu*(+) and ER(–), were invasive and metastatic in vivo and were highly positive for uPAR protein and uPA activity (Table 1). In contrast, the MCF-7 and T-47D cell lines had a more epithelial morphology, contained little or no EGFR/*neu* protein, were ER(+) and were neither invasive nor metastatic in vivo (although MCF-7 cells are known to form tumours in the presence of oestrogen; Thompson et al, 1992). Interestingly, the levels of plasma membrane and cell-surface uPAR protein were markedly reduced in the MCF-7 (four- to six- fold respectively) and even more so in T-47D (10- to 15-fold respectively) compared with levels in MDA-MB-231 cells. In agreement, a similar pattern was found for plasma membrane uPA activity (at least threefold for MCF-7 and 18-fold for T-47D) compared with plasma membrane levels in MDA-MB-231 cells. The results in Table 1 confirm that cells with a malignant phenotype are less differentiated and overexpress uPAR than the more differentiated and non-invasive cell lines.

**Figure 2** Plasminogen binding to MDA-MB-231 cells. (**A**) MDA-MB-231 cells were incubated with increasing concentrations of FITC-glu-plasminogen in the absence (X) or presence of 1 mM tranexamic acid (+) and analysed by dual-colour flow cytometry. The lysine-dependent plasminogen binding of viable cells (▲) was calculated as described in the legend to Figure 1. The values shown are means ± s.d. ($n$ = 3) from a representative experiment. (**B**) Representative Scatchard plot of lysine-dependent FITC-glu-plasminogen binding. This plot was derived from binding data generated by fluorimetry measurements and includes binding due to both viable and non-viable cells

### Cell-surface plasminogen binding on metastatic vs non-metastatic breast cancer cells

#### *Fluorescence studies*

The ability of plasminogen to interact with the cell surface was assessed by dual-colour flow cytometry. This technique was able to distinguish plasminogen binding to viable cells (cell-surface specific binding) from non-viable cells (binding to cell surfaces

© Cancer Research Campaign 1998

**Figure 3** Plasminogen ligand histochemistry of non-permeabilized breast cancer cells. Fixed and non-permeabilized MDA-MB-231 (**A–C**), MCF-7 (**D–F**) and T-47D (**G–I**) cells were incubated with biotinylated glu-plasminogen (0.2 μM) in the absence (**A**, **D**, **G**) or presence (**B**, **E**, **H**) of 5 mM tranexamic acid, followed by incubation with streptavidin–HRP. Lower panels (**C** ,**F**, **I**) show a lack of streptavidin–HRP interaction and subsequent colour reaction with cells that had been incubated with biotinylated BSA (0.2 μM). The arrow in panel (**D**) points to cellular debris and intracellular moieties). Density plots of fluorescence intensity due to FITC-glu-plasminogen binding vs propidium iodide uptake were used to establish 'gates' based on viability (Figure 1B; described in detail in Materials and methods). Cells that excluded the propidium iodide were gated as viable, whereas cells staining with propidium iodide were gated as non-viable (Figure 1B). Based on these parameters, fluorescence intensity due to cell-surface specific plasminogen binding only can be presented as histogram plots, as shown in Figure 1C. The histogram plots were used to calculate cell-surface lysine-dependent plasminogen binding (Figure 1A) and indicated that, while the MCF-7 and T-47D cell lines bound similarly low but detectable amounts of plasminogen (8.0 ± 0.8 and 9.5 ± 2.3 fluorescence units respectively), the MDA-MB-231 cell line bound significantly more plasminogen (21.2 ± 3.6 fluorescence units) than either the MCF-7 or the T-47D cell line.

Interestingly, the amount of FITC-glu-plasminogen binding to non-viable cells was consistently two orders of magnitude higher than to viable cells (refer to Figure 1B) and this was lysine dependent (data not shown). This phenomenon was also found in the MCF-7 and T-47D cell lines (data not shown).

Cell-surface plasminogen binding to MDA-MB-231 cells was lysine- and concentration-dependent, indicating a specific interaction (Figure 2A). At all plasminogen concentrations tested, binding to MCF-7 and T-47D cells was minimal compared with MDA-MB-231 cells (data not shown). The concentrations of plasminogen required to saturate binding [e.g. to saturate binding to pure recombinant α-enolase, at least 10 μM (~1 mg $ml^{-1}$) of plasminogen was necessary; Andronicos et al, 1997] were found to render most of the cells non-viable (data not shown). Thus, it was not possible to measure cell-surface plasminogen binding to viable cells at high concentrations. The reason for this apparent toxic effect is unclear, but it is unlikely to be due to a loss of membrane integrity resulting from enzymatic activity, as the binding experiments were all performed on ice. Despite this, the lysine-dependent plasminogen binding to MDA-MB-231 cells was

© Cancer Research Campaign 1998 

**Figure 4** Plasminogen ligand histochemistry of permeabilized breast cancer cells. Same as for legend to Figure 3 except that, after fixation, cells were permeabilized with Triton X-100 as described in Materials and methods further characterized by FITC-glu-plasminogen binding and fluorimeter analysis. This technique is similar in principle to radiolabelled plasminogen binding analysis techniques that allow quantitation of binding parameters by Scatchard analysis. However, these techniques cannot take into account any binding as a result of even a small percentage of non-viable cells, which are invariably present in most cell preparations, regardless of the care taken to prevent cell damage. Nevertheless, when lysine-dependent plasminogen binding curves were transformed into Scatchard plots, the best fit for the data was curvilinear (Figure 2B), indicating two classes of binding sites. The $K_d$ values for plasminogen binding to MDA-MB-231 cells were $1.8 \pm 0.6 \times 10^{-6}$ M for the higher affinity site and $2.0 + 0.9 \times 10^{-4}$ M for the lower affinity site. The numbers of binding sites per cell were $5.0 \pm 1.6 \times 10^{7}$ and $3.9 \pm 1.8 \times 10^{9}$ for the higher and lower affinity sites respectively. This difference in number of binding sites per cell (two orders of magnitude) is comparable to the difference in capacity seen on viable and non-viable cells by flow cytometry (see above and refer to Figure 1B) and suggests that the lower affinity sites are attributable to non-viable cell plasminogen binding.

### *Ligand histochemistry studies*

The plasminogen ligand histochemistry technique allowed plasminogen binding capacity to be visualized on viable cells that were attached and spread onto a substrata. The differences seen by flow cytometry between the cell lines (Figures 1 and 2) were reproduced by plasminogen ligand histochemistry with non-permeabilized cells (Figure 3). The highest amount of positive brown staining was seen in the MDA-MB-231 cell line (Figure 3A) compared with the MCF-7 (Figure 3D) or T-47D (Figure 3G) cell lines. In the presence of excess tranexamic acid plasminogen binding was greatly reduced in all three cell lines (Figure 3B, E and H), confirming that the cell lines bound plasminogen in a lysine-dependent manner. The ligand histochemistry technique also showed that, while staining was diffuse over the cell surfaces, the degree of plasminogen binding within each cell line was heterogeneous. This was especially apparent in the MCF-7 and T-47D cell lines, in which some cells showed distinctive staining while others appeared to be completely negative (Figure 3D and G).

Deliberate permeabilization of the cells resulted in an enhancement of the amount of plasminogen binding that could be

 © Cancer Research Campaign 1998

**Figure 5** Plasminogen ligand blotting of breast cancer cell whole-cell lysates. Whole-cell lysates (15 μg per lane) and human recombinant α-enolase (r-α-enolase, 5 μg per lane) were separated by 12% SDS-PAGE under reducing conditions and either stained with Coomassie Blue (**A**) or transferred and subjected to ligand blotting using 5 nM glu-plasminogen in the absence (**B**) or presence of 100 mM ε-amino-n-caproic acid (**C**). Blots (**B**) and (**C**) were derived from gels run, transferred and probed in parallel and were exposed onto the same piece of autoradiograph film so that a direct comparison could be made between them. Glu-plasminogen binding proteins were detected using an anti-plasminogen polyclonal antibody. The arrows point to bands with apparent molecular masses of 50 kDa and 30 kDa

**Figure 6** Plasminogen ligand blotting of breast cancer cell plasma membranes. Same as for legend to Figure 5 except that plasma membrane fractions (15 μg per lane) were used instead of whole-cell lysates substantially reduced in the presence of tranexamic acid in all three breast cancer cell lines (Figure 4). The distribution of staining in these cells appeared to be diffuse in the cytoplasm and on the cell surface, and was not associated with the nucleus. The results in Figure 4 suggest that permeabilization allows plasminogen to bind to intracellular moieties not otherwise available at the cell surface and that there are many more plasminogen binding intracellular moieties than cell surface ones in all the cell lines. These ligand histochemistry data correlated well with the flow cytometry data, which demonstrated that plasminogen binding was substantially greater in non-viable cells than in viable cells (refer to Figure 1B). Interestingly, while the permeabilized MDA-MB-231 cells (Figure 4A) still appeared to bind more plasminogen than the permeabilized MCF-7 (Figure 4D) and T-47D (Figure 4F) cell lines, this difference between the three cell lines was not as apparent as in the non-permeabilized cells (Figure 3). This may be as a result of the sheer magnitude of plasminogen binding in permeabilized cells, which has the effect of diminishing the differences in plasminogen binding capacity between the cell lines.

### Total cellular and membrane-associated plasminogen binding proteins in the three breast cancer cell lines

Several bands were detected in ligand blots of whole-cell lysates from the breast cancer cell lines (Figure 5). Of these, two were major plasminogen binding bands (apparent molecular masses 50 kDa and

**Figure 7** Plasmin formation by breast cancer cells. Breast cancer cell plasma membrane preparations (2 μg) were preincubated with 0.5 μM plasminogen for 30 min at room temperature, followed by a 5-min incubation in the absence (■) or presence (▨) of 1 mM aprotinin. Plasmin activity was then measured at 37°C over 20 min using the Spectrozyme PL substrate (0.25 mM final concentration). Plasmin activity in the absence of plasminogen and aprotinin was also measured (□). The inset shows specific FITC-aprotinin binding to viable MDA-MB-231 cells as assessed by dual-colour flow cytometry (refer to Figure 1B). The specific binding was calculated by subtracting binding in the presence of a 50-fold excess of unlabelled aprotinin from the total binding. The values shown are means ± s.d. ($n$ = 3) from representative experiments

30 kDa) and were present at similar levels in all three cell lines (Figure 5B). These bands corresponded to plasminogen binding proteins because the lysine analogue EACA completely abolished the binding of plasminogen to these proteins (Figure 5C).

Ligand blots of plasma membrane proteins indicated a distinct plasminogen binding protein profile on each of the three cell lines (Figure 6). The MCF-7 plasma membranes contained three major bands with apparent molecular masses of 57 kDa, 47 kDa and 33 kDa, as well as two minor bands of 40 kDa and 36 kDa (Figure 6B). The 47-kDa and the 33-kDa bands in the MCF-7 plasma membranes (Figure 6B) corresponded to the 50-kDa and 30-kDa bands, respectively, in the MCF-7 whole-cell lysate (Figure 5). While the MDA-MB-231 and T-47D plasma membranes did not contain detectable amounts of the 57-kDa or 47- to 50-kDa bands seen in the MCF-7 plasma membranes, the 30- to 33-kDa band was present in all plasma membranes as a major band (Figure 6B). The MDA-MB-231 plasma membranes contained two other major bands with apparent molecular masses of 36 kDa and 26 kDa (Figure 6B). The T-47D plasma membranes also contained the 36-kDa band but in lower concentration than in the MDA-MB-231 plasma membranes (Figure 6B). In all ligand blots the plasminogen binding protein r-α-enolase (Andronicos et al, 1997) was used as a positive control. In each case the presence of EACA inhibited plasminogen binding to the protein (Figures 5C and 6C).

### Plasmin generation is associated with high plasminogen binding capacity and uPA activity

The ability to generate plasma membrane-associated plasmin activity was compared between the three breast cancer cell lines (Figure 7). Not surprisingly, the MDA-MB-231 cells, which have the highest plasminogen binding capacity and highest endogenous uPA activity, were highly efficient at generating plasmin. This activity was dependent on the presence of plasminogen and was completely inhibited by preincubation with the high-affinity plasmin inhibitor aprotinin. At a plasminogen concentration of 0.5 μM, there was no detectable plasmin specific activity in either the MCF-7 or the T-47D plasma membranes compared with 110 pmol min$^{-1}$ mg$^{-1}$ in the MDA-MB-231 plasma membranes. At higher concentrations of plasminogen and membrane protein, a small amount of plasmin specific activity could be detected in the MCF-7 and T-47D plasma membranes, however this was minimal compared with that detected for MDA-MB-231 plasma membranes (data not shown).

In order to show that plasmin was generated on the surfaces of viable MDA-MB-231 cells, dual-colour flow cytometry was used to establish 'gates' based on viability (refer to Figure 1B and Materials and methods) using FITC-aprotinin as the ligand. The use of FITC-aprotinin as a specific detector of plasmin activity had been previously described (Ellis et al, 1987). The inset to Figure 7 show that FITC-aprotinin bound to viable cells but only when preincubated with plasminogen, suggesting that MDA-MB-231 cells were capable of activating receptor-bound plasminogen on the cell surface.

### DISCUSSION

This study demonstrates that the spindle-shaped, EGFR (+)/*eu*(+), ER(–) and metastatic MDA-MB-231 cells were not only associated with high uPA activity and uPAR overexpression but that they also had an increased capacity to bind and activate cell-surface plasminogen compared with the more differentiated, non-metastatic cell lines MCF-7 and T-47D. Our results suggest that expression of cell-surface plasminogen receptors plays a part in regulating the plasminogen activation cascade and may contribute to the metastatic phenotype of the MDA-MB-231 cell line.

Dual-colour flow cytometry was used initially as a means to identify cell surface-specific plasminogen binding. However, this technique also revealed that both viable and non-viable cells could bind FITC-glu-plasminogen in a lysine-dependent manner. Moreover, non-viable cells bound two orders of magnitude more plasminogen than viable cells. This was confirmed by ligand histochemistry, which showed that biotinylated plasminogen binding capacity was substantially increased in all cell lines after permeabilization. This is an important finding as it is very difficult to harvest adherent cells and maintain them throughout experimental procedures at 100% viability (we consistently found that 10%, sometimes up to 20%, of the cells were non-viable as assessed by propidium iodide uptake by the time cells were analysed by flow cytometry) and suggests that even a small proportion of non-viable cells could affect evaluation of authentic cell-surface plasminogen binding capacity if viability status is not considered.

Scatchard plots of plasminogen binding data to MDA-MB-231 cells using a single-colour fluorescence technique resulted in curvilinear plots that are suggestive of two classes of binding sites.

© Cancer Research Campaign 1998

Curvilinear Scatchard plots of plasminogen binding have been reported previously for endothelial cells using a radiolabelled plasminogen binding technique (Ganz et al, 1991). The higher-affinity binding site in the MDA-MB-231 cells had an average $K_d$ of 1.8 μM and $5.0 \times 10^7$ binding sites per cell, while the lower affinity one had an average $K_d$ of 200 μM and $3.9 \times 10^9$ binding sites per cell. The affinity and capacity of the higher-affinity site are similar to that reported for many cell types determined by radiolabelled glu-plasminogen techniques (Plow and Miles, 1990; Hembrough et al, 1995) and are within physiological limits as plasminogen is found in the circulation at 2 μM. The capacity of the higher-affinity site is two orders of magnitude higher than the lower-affinity site – exactly the difference seen in capacity between viable and non-viable cells by dual-colour flow cytometry. As non-viable cells were present in all fluorimetry experiments (up to 15% of total cell sample), it is likely that the lower-affinity, very-high-capacity binding sites are due to non-viable cells. The affinity and capacity of these non-viable cells implies that it would be hard to saturate binding. Indeed the non-viable cell binding curves (generated by dual-colour flow cytometry; data not shown) were linear at the concentrations of plasminogen used for assessing cell-surface binding (refer to Figure 2A). Nevertheless, while binding to non-viable cells may not be physiologically relevant, taken together, our results suggest that plasminogen binding to non-viable cells is so large that subtle or even significant differences in cell surface-specific plasminogen binding capacity between cells can be missed when measured and analysed with techniques that cannot distinguish between viable and non-viable cells.

Our results also suggest that there are many more intracellular plasminogen binding moieties than cell surface ones, regardless of the cell-surface binding capacity. The 50-kDa plasminogen binding protein present in the whole-cell lysates of MDA-MB-231, MCF-7 and T-47D cells may account for at least some of the increase in plasminogen binding capacity of both the MDA-MB-231 and the T-47D cell line upon permeabilization. While all of the cell lines expressed plasma membrane-associated plasminogen binding proteins, the plasminogen ligand blots gave no information about the orientation of plasminogen binding proteins within the plasma membranes of intact cells. Therefore, the presence of these proteins in the plasma membrane does not necessarily mean that they would be oriented on the cell surface in such a way that they could bind pericellular plasminogen. As viable or non-permeabilized MCF-7 and T-47D cells had minimal cell-surface plasminogen binding capacity compared with viable or non-permeabilized MDA-MB-231 cells, perhaps only a small proportion of the MCF-7 and T-47D plasma membrane-associated proteins are physiologically available to bind extracellular plasminogen. Upon permeabilization a greater proportion of these proteins may also become available for plasminogen binding in the MCF-7 and T-47D cells. It is also possible that non-proteinaceous binding moieties, such as gangliosides (Miles et al, 1989), may account for a significant proportion of the differences in cell-surface plasminogen binding between the cell lines.

Nevertheless, the ligand blot data indicate that more than one protein moiety contributes to total cellular plasminogen binding capacity in these cell lines. The intermediate filament protein cytokeratin 8 and the glycolytic enzyme α-enolase are two potentially important receptors that have been isolated from the plasma membranes of rat hepatocytes (Hembrough et al, 1995), the lymphoid monocytic cell line U-937 (Miles et al, 1991) and embryonic rat neurons (Nakajima et al, 1994). Cytokeratin 8 has been localized to the cell surface of human breast cancer cells (Hembrough et al, 1995, 1996), and an α-enolase-like protein with plasminogen binding ability was isolated from total cell lysates of two human breast cancer cell lines (Lopez-Alemany et al, 1994). We have previously demonstrated that the glycolytic enzyme α-enolase is an authentic plasminogen binding protein and that it is present as a 47-kDa protein in whole-cell lysates of various human cancer cell lines, including the breast cancer cell lines used in this study (Andronicos et al, 1997). Using a [$^{125}$I]plasminogen overlay assay, Hembrough et al (1996) showed the presence of several plasminogen binding proteins in the cytoplasm and plasma membrane fractions of various breast cancer cells. Moreover, they showed the presence of a major 55-kDa plasma membrane-associated plasminogen binding protein in MCF-7 cells, which they identified as cytokeratin 8. Thus, it is possible that the 47- to 50-kDa and the 57-kDa plasminogen binding proteins detected in the current study may be α-enolase and cytokeratin 8 respectively. The possible identity of the other plasminogen binding proteins remain unclear. However, the molecular mass of the 30- to 33-kDa protein is similar to that of another plasminogen binding protein, amphoterin (Parkkinen and Rauvala, 1991).

Thus it is clear that a number of proteinaceous and possibly non-proteinaceous moieties are likely to contribute to cell-surface plasminogen binding. It is therefore difficult to design specific experiments aimed at establishing a direct relationship between plasminogen binding capacity and metastatic potential without identifying all of the plasminogen binding moieties on the breast cancer cell lines. However, Stonelake et al (1997) recently demonstrated that, in the presence of plasminogen, MDA-MB-231 and other metastatic breast cancer cell lines, unlike non-metastatic cell lines, such as MCF-7 and T-47D, had the ability to degrade human endothelial basement membrane and that this activity was significantly inhibited by specific uPA or plasmin inhibitors. These authors also demonstrated a similar inhibitory effect by lysine analogues and attributed this result to an inhibition of plasminogen binding. These results strongly corroborate our data, which indicates that the plasminogen binding capacity of breast cancer cells modulates plasmin activity in the presence of uPA and, taken together, establishes a relationship between the plasminogen binding capacity of breast cancer cells and their metastatic potential, at least in an in vitro model.

The differences in cell-surface plasminogen binding between the metastatic MDA-MB-231 cells vs the non-metastatic MCF-7 and T-47D cells are possibly related to the ability of cells to target some or all of their plasminogen binding proteins to the cell surface. One possible mechanism may be linked to the difference in uPA/uPAR status between the cell lines. Incubation of the WISH epithelial cell line, which expresses high levels of uPAR but undetectable levels of uPA, with inactive uPA resulted in the phosphorylation and redistribution of the cytoskeletal components cytokeratin 8 and 18 (Busso et al, 1994). From this, it was suggested that signal transduction pathways via the uPAR GPI anchor are involved in cell migration (Busso et al, 1994). As cytokeratin 8 has been shown to be a plasminogen receptor associated with the external surfaces of human breast cancer cells (Hembrough et al, 1995, 1996), signal transduction events associated with uPAR expression may be one mechanism that leads to an increased capacity of breast cancer cells, such as the MDA-MB-231 cells, to bind cell-surface plasminogen.

It is conceivable that variations in the activity of intracellular signalling proteins due to EGFR and *erb*B-2/*neu* expression may

© Cancer Research Campaign 1998

initiate events that lead to the translocation of proteins within the cell (Milligan et al, 1995) and may in some way affect the localization of proteins that can act as plasminogen receptors if placed in the correct orientation at the cell surface. Amino-or carboxy-terminal lysines appear to be the only feature common and necessary to all candidate plasminogen binding proteins. Any intracellular protein with an amino- or carboxy-terminal lysine that is also subject to the above modifications could be translocated to the outer surface of the plasma membrane and act as a plasminogen receptor.

Other markers whose cellular expression correlates with metastatic potential have been identified in human breast cancer cell lines. These include matrix metalloproteinase-2 (Azzam et al, 1993), vimentin (Thompson et al, 1992) and surface glycoproteins such as CD44 (Culty et al, 1994). However, a review of the literature indicates that overexpression of components of the plasminogen activation cascade play an important role in breast cancer invasion and metastasis. While stromal cells adjacent to cancer cells in breast cancer tissue may contribute to invasion, by expressing uPA and/or uPAR for example (Christensen et al, 1996; Costantini et al, 1996; Nielsen et al, 1996), our data and that of others (Holst-Hansen et al, 1996; Stonelake et al, 1997) clearly show that breast epithelial cells with a metastatic phenotype have the capacity to efficiently convert plasminogen to plasmin. A combination of studies suggests that the concentration and spatial distribution of the combination of uPA, uPAR, PAI-1 and PAI-2 expression in human breast carcinomas allow a better indication of degree of malignancy (Foekens et al, 1995; Christensen et al, 1996; Costantini et al, 1996). The results presented in this paper strongly suggest that another component of the plasminogen activation cascade, i.e. plasminogen receptors, is important and their role in human breast cancer should be further characterized.

### ACKNOWLEDGEMENTS

This study was supported by project grants nos. 940857 and 970808 from the National Health and Medical Research Council of Australia. NMA was a recipient of an American Diagnostica–University of Wollongong PhD Scholarship. Oestrogen receptor immunohistochemistry was kindly performed by Southern Pathology, Wollongong, Australia.

### REFERENCES

Andronicos NM, Ranson M, Bognacki J and Baker MS (1997) The human ENO1 gene product (recombinant human α-enolase) displays characteristics required for a plasminogen binding protein. *Biochim Biophys Acta* **1337**: 27–39

Azzam HS, Arand G, Lippman ME and Thompson EW (1993) Association of MMP-2 activation potential with metastatic progression in human breast cancer cell lines independent of MMP-2 production. *J Natl Cancer Inst* **85**: 1758–1764

Baker MS, Bleakley PA, Woodrow G and Doe WF (1990) Inhibition of cancer cell urokinase plasminogen activator by its specific inhibitor PAI-2 and subsequent effects on extracellular matrix degradation. *Cancer Res* **50**: 4676–4684

Burtin P, Zhang S, Schauffler J, Komano O, Sastre X and Mathieu MC (1993) Visualization of the plasmin receptor on sections of human mammary carcinoma cells. *Int J Cancer* **53**: 17–21

Busso N, Masur SK, Lazega D, Waxman S and Ossowski L (1994) Induction of cell migration by pro-urokinase binding to its receptor: possible mechanism for signal transduction in human epithelial cells. *J Cell Biol* **126**: 259–270

Castellino FJ (1995) Plasminogen. In *Molecular Basis of Thrombosis and Hemostasis*, High KA and Roberts HR. (eds), pp. 495–515. Marcel Dekker: New York NY

Cesarman GM, Guevara CA and Hajjar KA (1994) An endothelial cell receptor for plasminogen/tissue plasminogen activator (t-PA). II. Annexin II-mediated enhancement of t-PA-dependent plasminogen activation. *J Biol Chem* **269**: 21198–21203

Christensen L, Wiborg-Simonsen AC, Heegaard CW, Moestrup SK, Andersen JA and Andreasen PA (1996) Immunohistochemical localization of urokinase-type plasminogen activator inhibitor, urokinase receptor, and alpha(2)-macroglobulin receptor in human breast carcinomas. *Int J Cancer* **66**: 441–452

Coleman PL and Green GDJ (1981) A sensitive coupled assay for plasminogen activator using a thiol ester substrate for plasmin. *Proc Natl Acad Sci* USA **370**: 617–626

Connolly JM and Rose DP (1997) Expression of the invasive phenotype by MCF-7 human breast cancer cells transfected to overexpress protein kinase C-alpha or the erbB2 proto-oncogene. *Int J Oncol* **10**: 71–76

Costantini V, Sidoni A, Deveglia R, Cazzato OA, Bellezza G, Ferri I, Bucciarelli E and Nenci GG (1996) Combined overexpression of urokinase, urokinase receptor, and plasminogen activator inhibitor-1 is associated with breast cancer progression: an immunohistochemical comparison of normal, benign, and malignant breast tissues. *Cancer* **77**: 1079–1088

Culty M, Shizari M, Thompson EW and Underhill CB (1994) Binding and degradation of hyaluronan by human breast cancer cell lines expressing different forms of CD44: correlation with metastatic potential. *J Cell Physiol* **160**: 275–286

Darzynkiewicz Z, Li X and Gong J (1994) Analysis of cell viability: discrimination of cells dying by apoptosis. In *Methods in Cell Biology*, Vol. 41. pp. 18–22. Academic Press

De Vries TJ, De Wit PEJ, Clemmensen I, Verspaget HW, Weidle UH, Brocker EB, Ruiter DJ and van Muijen GNP (1996) Tetranectin and plasmin/plasminogen are similarly distributed at the invasive front of cutaneous melanoma lesions. *J Pathol* **179**: 260–265

Duffy MJ (1993) Urokinase-plasminogen activator and malignancy. *Fibrinolysis* **7**: 295–302

Ellis V, Scully MF and Kakkar VV (1987) Plasminogen activation by single-chain urokinase in functional isolation. *J Biol Chem* **262**: 14998–15003

Evans CW (1991) *The Metastatic Cell: Behaviour and Biochemistry*. Chapman and Hall: London

Foekens JA, Buessecker F, Peters HA, Krainick U, Van Putten WLJ, Look MP, Klijn JGM and Kramer MD (1995) Plasminogen activator inhibitor-2: prognostic relevance in 1012 patients with primary breast cancer. *Cancer Res* **55**: 1423–1427

Ganz PR, Dupuis D, Dudani AK and Hashemi S (1991) Characterization of plasminogen binding to human capillary and arterial endothelial cells. *Biochem Cell Biol* **69**: 442–448

Goding JW (1976) Conjugation of antibodies with fluorochromes: modification to the standard methods. *J Immunol Methods* **13**: 215–226

Harlow E and Lane D (1988) *Antibodies: A Laboratory Manual*. pp. 386–387. Cold Spring Harbor Laboratory.

Hembrough TA, Vasudevan J, Allietta MM, Glass WF and Gonias SL (1995) A cytokeratin 8-like protein with plasminogen-binding activity is present on the external surfaces of hepatocytes, HepG2 cells and breast carcinoma cell lines. *J Cell Science* **108**: 1071–1082

Hembrough TA, Li L and Gonias SL (1996) Cell-surface cytokeratin 8 is the major plasminogen receptor on breast cancer cells and is required for the accelerated activation of cell-associated plasminogen by tissue-type plasminogen activator. *J Biol Chem* **271**: 25684–25691

Holst-Hansen C, Johannessen B, Hoyer-Hansen G, Romer J, Ellis V and Brunner N (1996) Urokinase-type plasminogen activation in three human breast cancer cell lines correlates with their in vitro invasiveness. *Clin Exp Metastasis* **14**: 297–307

Janicke F, Schmitt M and Graeff H (1991) Clinical relevance of the urokinase-type and tissue-type plasminogen activators, and of their type 1 inhibitor in breast cancer. *Semin Thromb Hemostasis* **17**: 303–312

Kook YH, Adamski J, Zelent A and Ossowski L (1994) The effect of antisense inhibition of urokinase receptor in human squamous cell carcinoma on malignancy. *EMBO J* **17**: 3983–3991

Lee CSL, Hall RL, Alexander IE, Koga M, Shine J and Sutherland RL (1990) Inverse relationship between estrogen receptor and epidermal growth factor receptor mRNA levels in human breast cancer cell lines. *Growth Factors* **3**: 97–103

Long BJ and Rose DP (1996) Invasive capacity and regulation of urokinase-type plasminogen activator in estrogen receptor (ER)-negative MDA-MB-231 human breast cancer cells, and a transfectant (S30) stably expressing ER. *Cancer Lett* **99**: 209–215

Lopez-Alemany R, Correc P, Camoin L and Burtin P (1994) Purification of the plasmin receptor from human carcinoma cells and comparison to α-enolase. *Thromb Res* **75**: 371–381

© Cancer Research Campaign 1998

Mignatti P and Rifkin DB (1993) Biology and biochemistry of proteinases in tumor invasion. *Physiol Rev* **73**: 161–195

Miles LA, Dahlberg CM, Levin EG and Plow EF (1989) Gangliosides interact directly with plasminogen and urokinase and may mediate binding of these fibrinolytic components to cells. *Biochemistry* **28**: 9337–9343

Miles LA, Dahlberg CM, Plescia J, Felez J, Kato K and Plow EF (1991) Role of cell-surface lysines in plasminogen binding to cells: identification of α-enolase as a candidate plasminogen receptor. *Biochemistry* **30**: 1682–1691

Milligan G, Parenti M and Magee AI (1995) The dynamic role of palmitoylation in signal transduction. *TIBS* **20**: 181–186

Moller LB (1993) Structure and function of the urokinase receptor. *Blood Çoagul Fibrinol* **4**: 293–303

Nakajima K, Hamanoue M, Takemoto N, Hattori T, Kato K and Kohsaka S (1994) Plasminogen binds specifically to α-enolase on rat neuronal plasma membrane. *J Neurochem* **63**: 2048–2057

Nielsen BS, Sehested M, Timshel S, Pyke C and Dano K (1996) Messenger RNA for urokinase plasminogen activator is expressed in myofibroblasts adjacent to cancer cells in human breast cancer. *Lab Invest* **74**: 168–177

Parkkinen J and Rauvala H (1991) Interactions of plasminogen and tissue plasminogen activator (t-PA) with amphoterin. Enhancement of t-PA-catalysed plasminogen activation by amphoterin. *J Biol Chem* **266**: 16730–16735

Plow EF and Miles LA (1990) Plasminogen receptors in the mediation of pericellular proteolysis. *Cell Diff Devel* **32**: 293–298

Rana APS and Majumder GC (1987) Factors influencing the yield and purity of goat sperm plasma membranes isolated by means of an aqueous two-phase polymer system. *Preparative Biochem* **17**: 261–281

Singleton TP and Strickler JG (1992) Clinical and pathological significance of the c-erbB-2 (HER-2/neu) oncogene. *Pathol Annu* **1**: 165–190

Stack MS, Moser TL and Pizzo SV (1992) Binding of human plasminogen to basement-membrane (type IV) collagen. *Biochem J* **284**: 103–108

Stonelake PS, Jones CE, Neoptolemos JP and Baker PR (1997) Proteinase inhibitors reduce basement membrane degradation by human breast cancer cells. *Br J Cancer* **75**: 951–959

Thompson EW, Paik S, Brunner N, Sommers CL, Zugmaier G, Clarke R, Shima T, Torri J, Donahue S, Lipmann ME, Martin GR and Dickson RB (1992) Association of increased basement membrane invasiveness with absence of estrogen receptor and expression of vimentin in human breast cancer cell lines. *J Cell Physiol* **150**: 534–544

© Cancer Research Campaign 1998


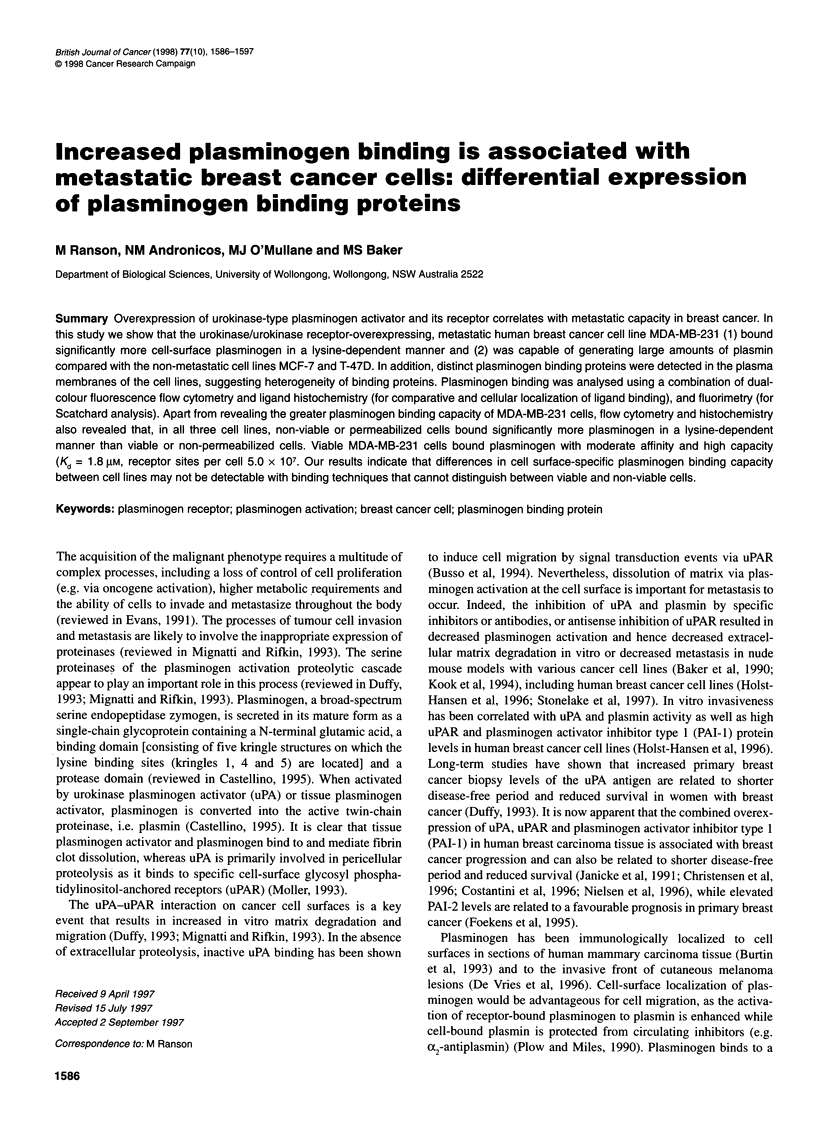

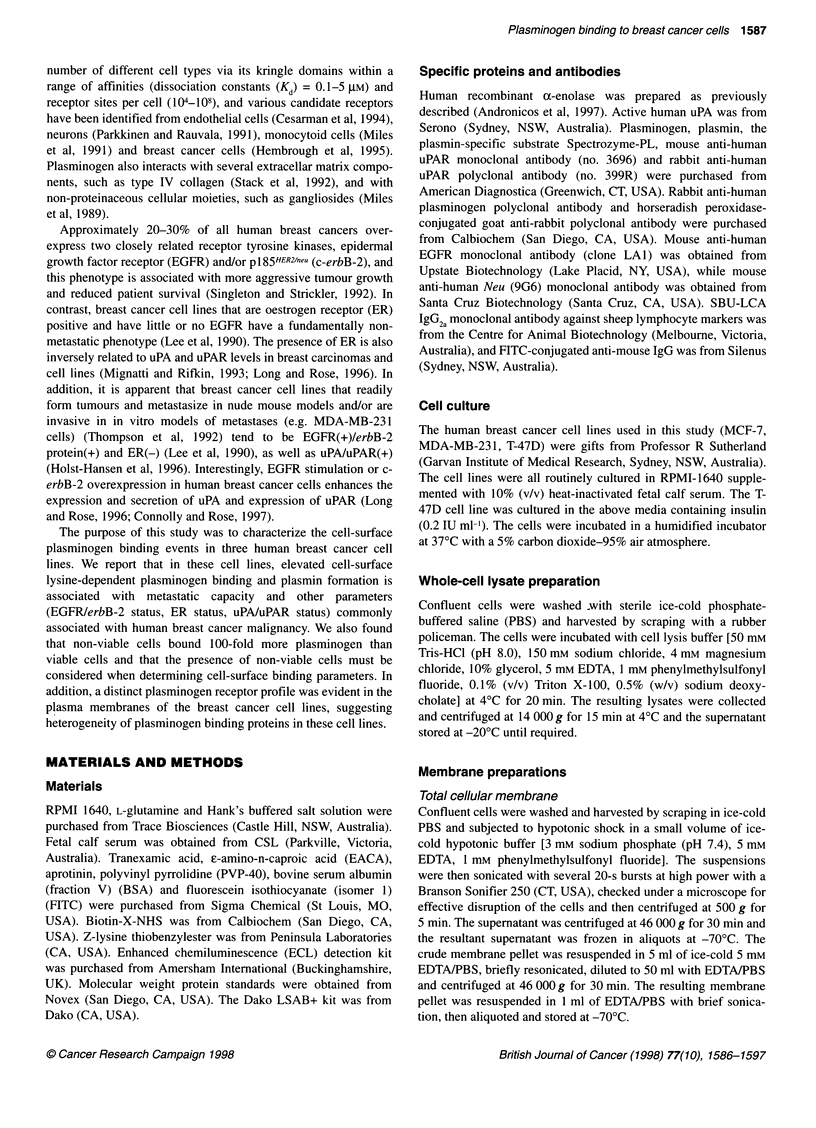

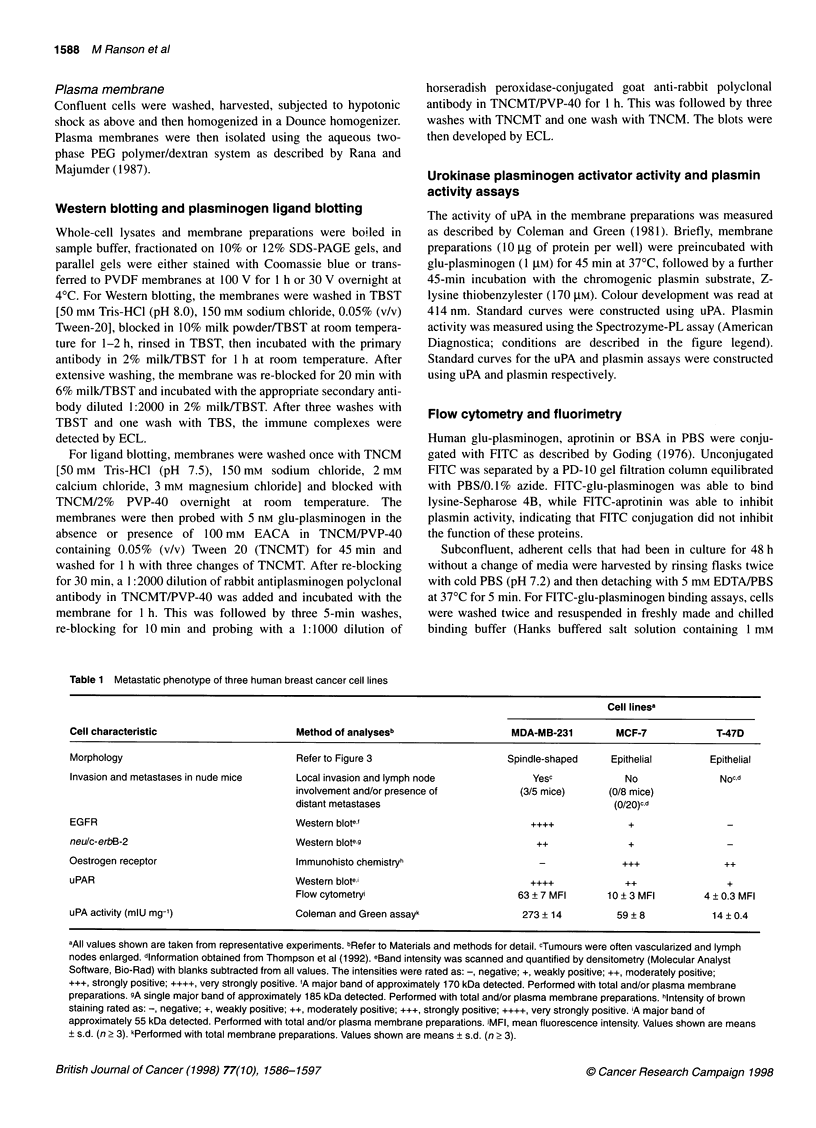

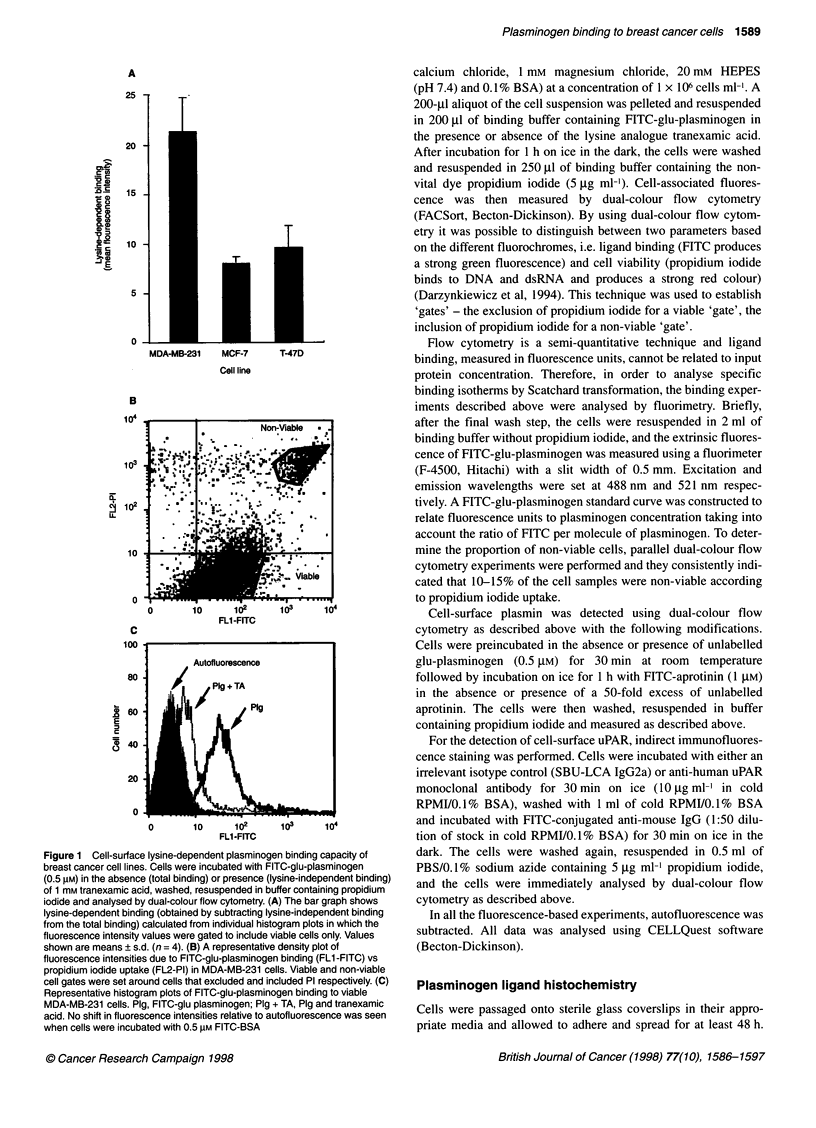

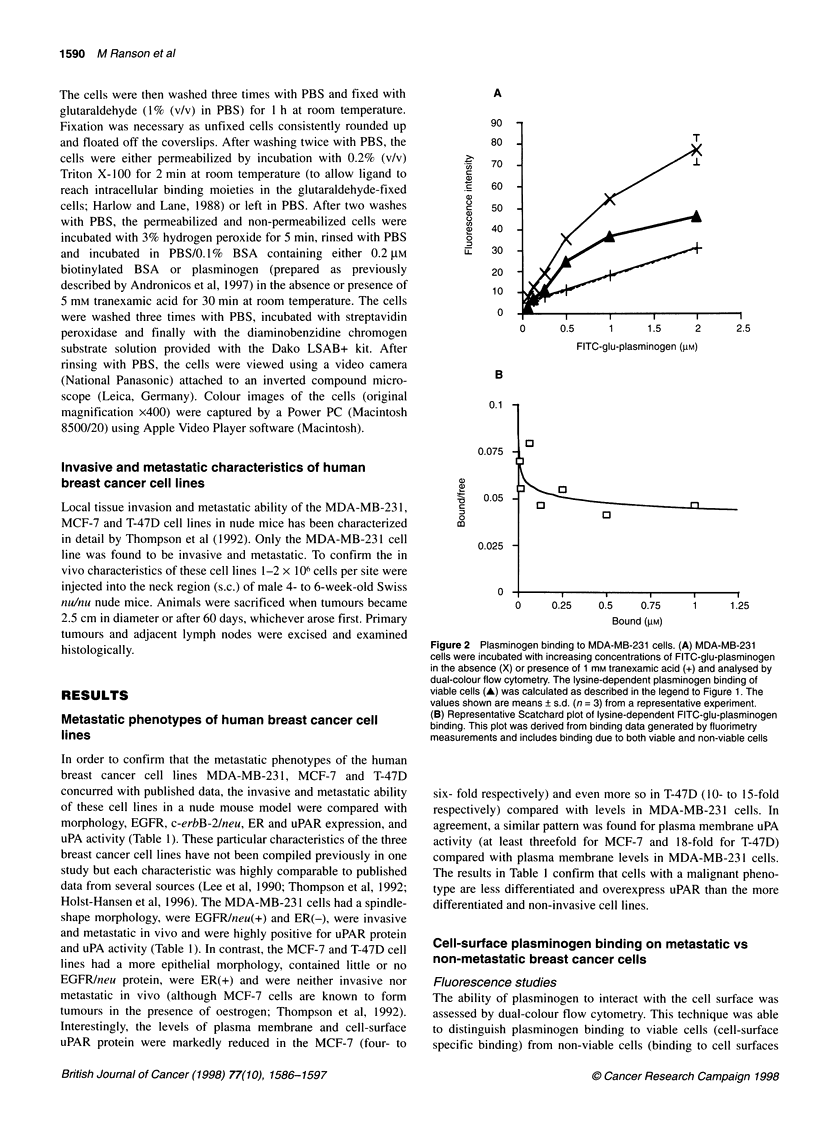

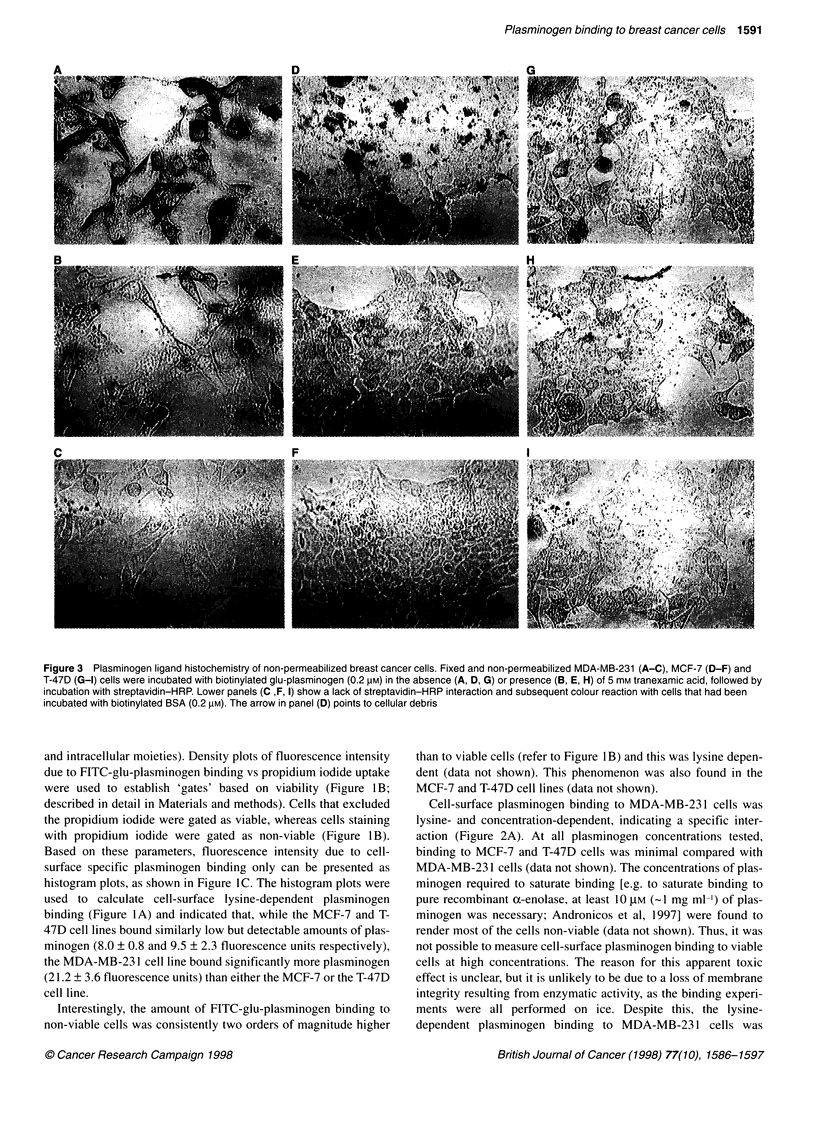

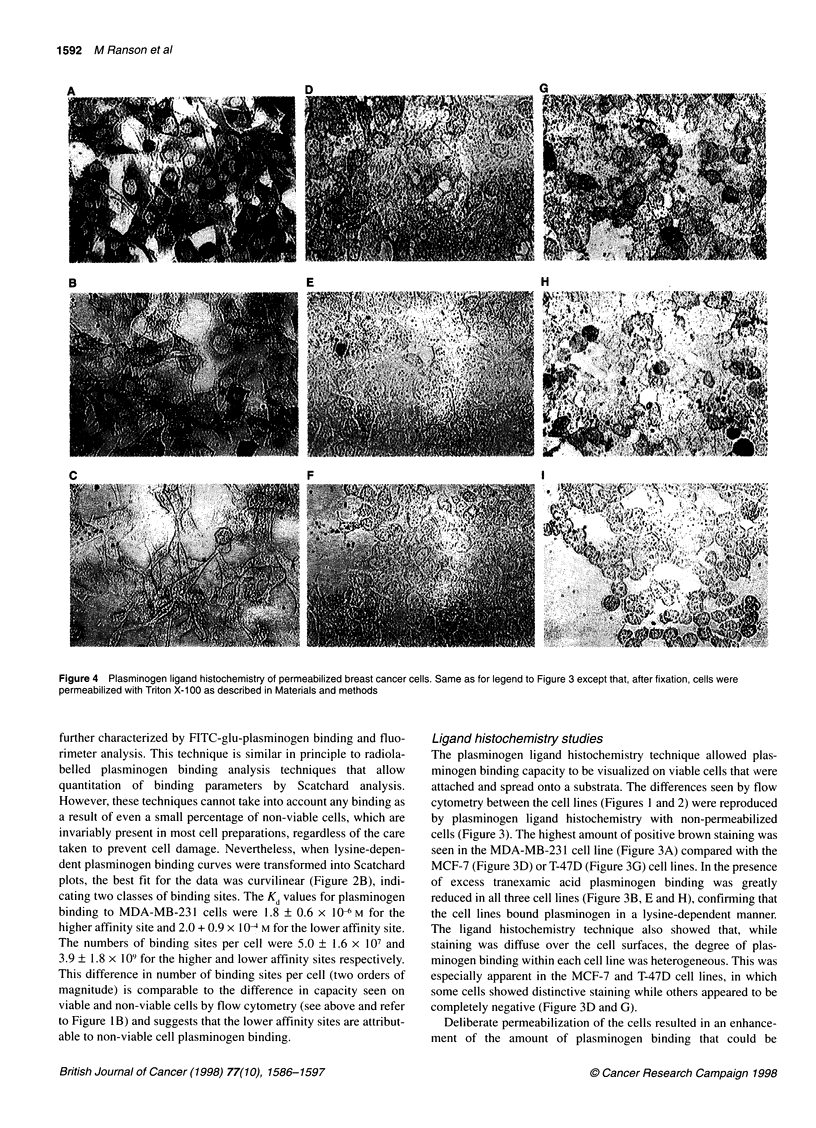

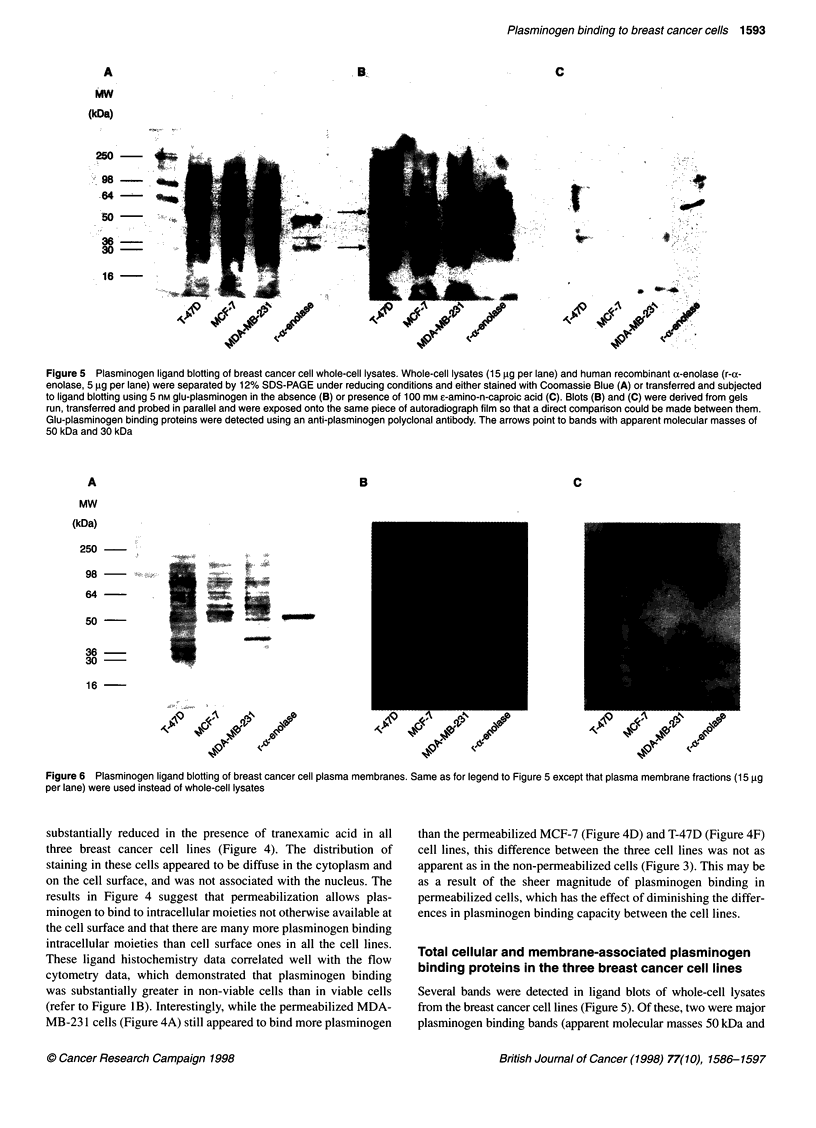

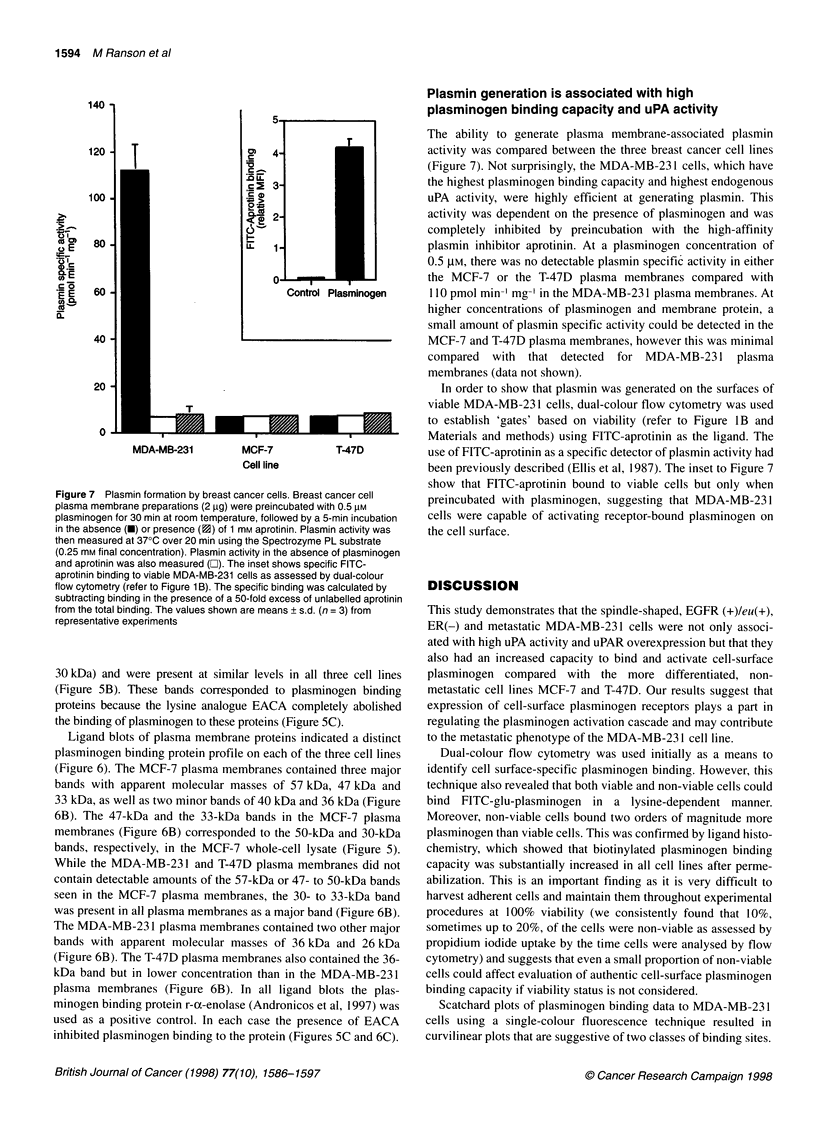

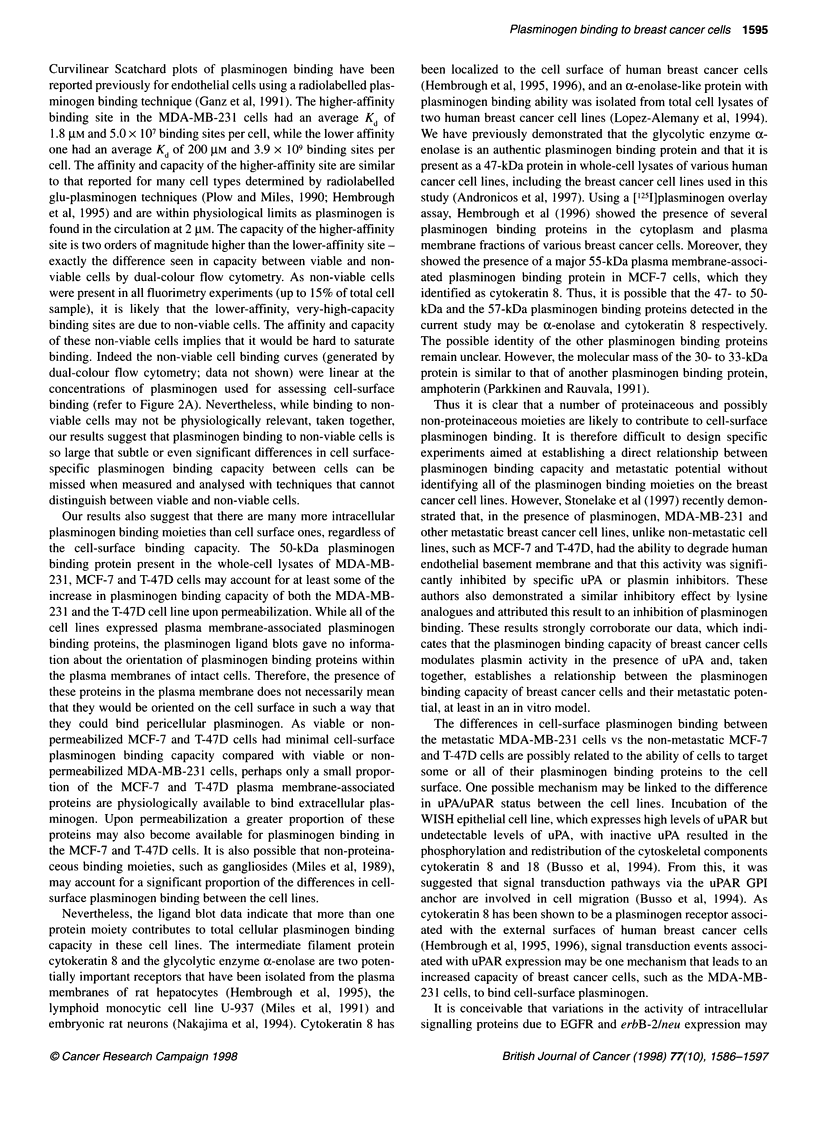

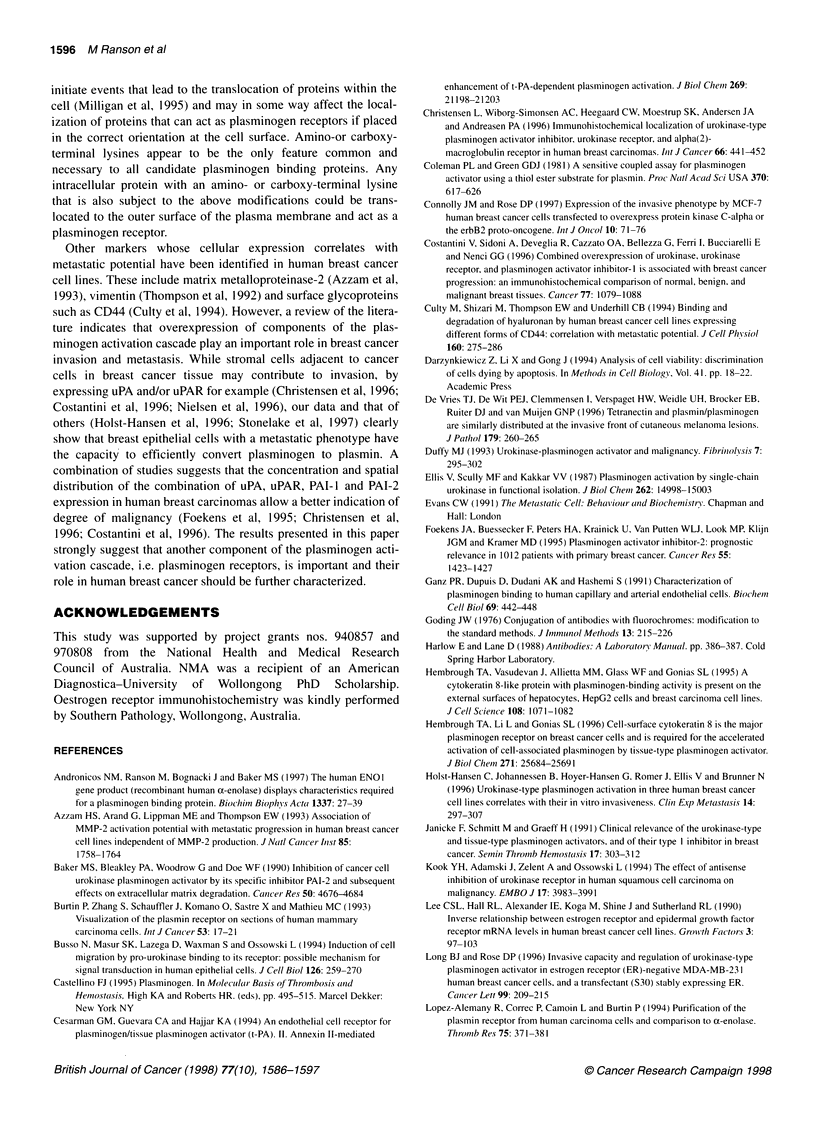

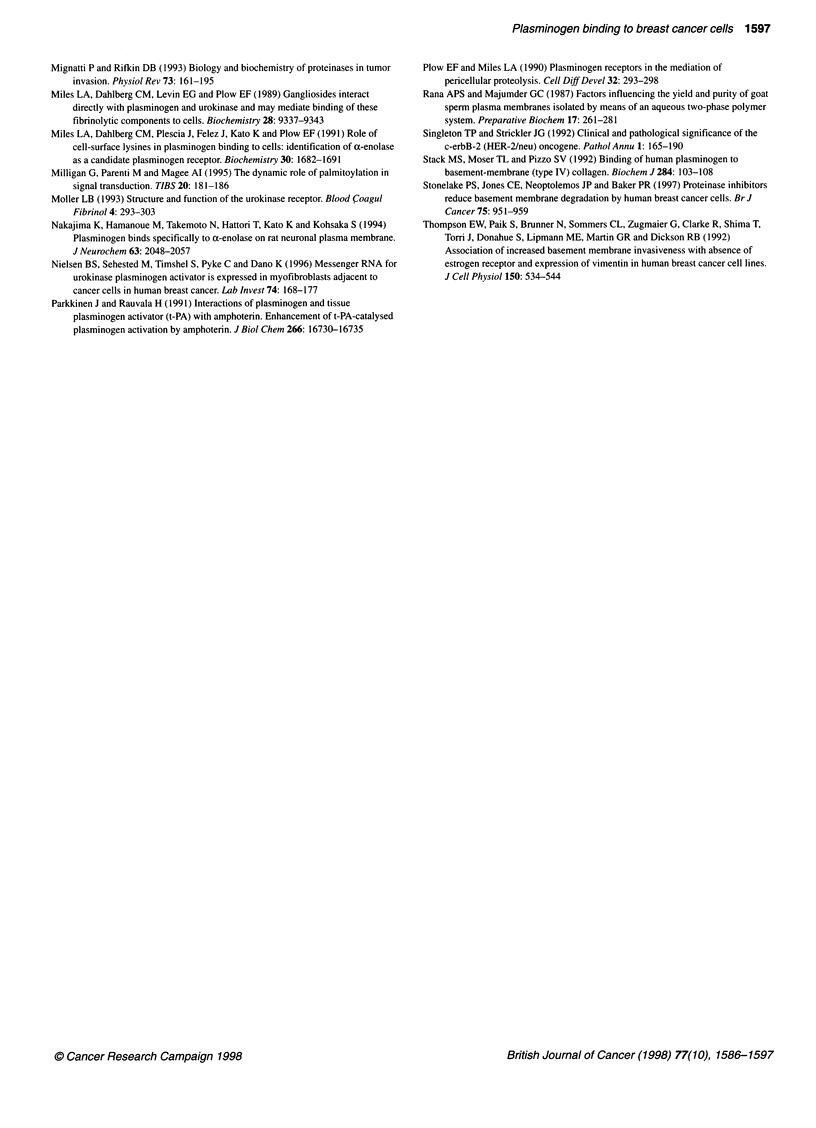


## References

[OCR_01270] Andronicos N. M., Ranson M., Bognacki J., Baker M. S. (1997). The human ENO1 gene product (recombinant human alpha-enolase) displays characteristics required for a plasminogen binding protein.. Biochim Biophys Acta.

[OCR_01275] Azzam H. S., Arand G., Lippman M. E., Thompson E. W. (1993). Association of MMP-2 activation potential with metastatic progression in human breast cancer cell lines independent of MMP-2 production.. J Natl Cancer Inst.

[OCR_01281] Baker M. S., Bleakley P., Woodrow G. C., Doe W. F. (1990). Inhibition of cancer cell urokinase plasminogen activator by its specific inhibitor PAI-2 and subsequent effects on extracellular matrix degradation.. Cancer Res.

[OCR_01286] Burtin P., Zhang S., Schauffler J., Komano O., Sastre X., Mathieu M. C. (1993). Visualization of the plasmin receptor on sections of human mammary carcinoma cells.. Int J Cancer.

[OCR_01291] Busso N., Masur S. K., Lazega D., Waxman S., Ossowski L. (1994). Induction of cell migration by pro-urokinase binding to its receptor: possible mechanism for signal transduction in human epithelial cells.. J Cell Biol.

[OCR_01300] Cesarman G. M., Guevara C. A., Hajjar K. A. (1994). An endothelial cell receptor for plasminogen/tissue plasminogen activator (t-PA). II. Annexin II-mediated enhancement of t-PA-dependent plasminogen activation.. J Biol Chem.

[OCR_01307] Christensen L., Wiborg Simonsen A. C., Heegaard C. W., Moestrup S. K., Andersen J. A., Andreasen P. A. (1996). Immunohistochemical localization of urokinase-type plasminogen activator, type-1 plasminogen-activator inhibitor, urokinase receptor and alpha(2)-macroglobulin receptor in human breast carcinomas.. Int J Cancer.

[OCR_01313] Coleman P. L., Green G. D. (1981). A sensitive, coupled assay for plasminogen activator using a thiol ester substrate for plasmin.. Ann N Y Acad Sci.

[OCR_01323] Costantini V., Sidoni A., Deveglia R., Cazzato O. A., Bellezza G., Ferri I., Bucciarelli E., Nenci G. G. (1996). Combined overexpression of urokinase, urokinase receptor, and plasminogen activator inhibitor-1 is associated with breast cancer progression: an immunohistochemical comparison of normal, benign, and malignant breast tissues.. Cancer.

[OCR_01331] Culty M., Shizari M., Thompson E. W., Underhill C. B. (1994). Binding and degradation of hyaluronan by human breast cancer cell lines expressing different forms of CD44: correlation with invasive potential.. J Cell Physiol.

[OCR_01353] Ellis V., Scully M. F., Kakkar V. V. (1987). Plasminogen activation by single-chain urokinase in functional isolation. A kinetic study.. J Biol Chem.

[OCR_01361] Foekens J. A., Buessecker F., Peters H. A., Krainick U., van Putten W. L., Look M. P., Klijn J. G., Kramer M. D. (1995). Plasminogen activator inhibitor-2: prognostic relevance in 1012 patients with primary breast cancer.. Cancer Res.

[OCR_01367] Ganz P. R., Dupuis D., Dudani A. K., Hashemi S. (1991). Characterization of plasminogen binding to human capillary and arterial endothelial cells.. Biochem Cell Biol.

[OCR_01372] Goding J. W. (1976). Conjugation of antibodies with fluorochromes: modifications to the standard methods.. J Immunol Methods.

[OCR_01386] Hembrough T. A., Li L., Gonias S. L. (1996). Cell-surface cytokeratin 8 is the major plasminogen receptor on breast cancer cells and is required for the accelerated activation of cell-associated plasminogen by tissue-type plasminogen activator.. J Biol Chem.

[OCR_01380] Hembrough T. A., Vasudevan J., Allietta M. M., Glass W. F., Gonias S. L. (1995). A cytokeratin 8-like protein with plasminogen-binding activity is present on the external surfaces of hepatocytes, HepG2 cells and breast carcinoma cell lines.. J Cell Sci.

[OCR_01392] Holst-Hansen C., Johannessen B., Høyer-Hansen G., Rømer J., Ellis V., Brünner N. (1996). Urokinase-type plasminogen activation in three human breast cancer cell lines correlates with their in vitro invasiveness.. Clin Exp Metastasis.

[OCR_01398] Jänicke F., Schmitt M., Graeff H. (1991). Clinical relevance of the urokinase-type and tissue-type plasminogen activators and of their type 1 inhibitor in breast cancer.. Semin Thromb Hemost.

[OCR_01403] Kook Y. H., Adamski J., Zelent A., Ossowski L. (1994). The effect of antisense inhibition of urokinase receptor in human squamous cell carcinoma on malignancy.. EMBO J.

[OCR_01408] Lee C. S., Hall R. E., Alexander I. E., Koga M., Shine J., Sutherland R. L. (1990). Inverse relationship between estrogen receptor and epidermal growth factor receptor mRNA levels in human breast cancer cell lines.. Growth Factors.

[OCR_01414] Long B. J., Rose D. P. (1996). Invasive capacity and regulation of urokinase-type plasminogen activator in estrogen receptor (ER)-negative MDA-MB-231 human breast cancer cells, and a transfectant (S30) stably expressing ER.. Cancer Lett.

[OCR_01420] Lopez-Alemany R., Correc P., Camoin L., Burtin P. (1994). Purification of the plasmin receptor from human carcinoma cells and comparison to alpha-enolase.. Thromb Res.

[OCR_01429] Mignatti P., Rifkin D. B. (1993). Biology and biochemistry of proteinases in tumor invasion.. Physiol Rev.

[OCR_01433] Miles L. A., Dahlberg C. M., Levin E. G., Plow E. F. (1989). Gangliosides interact directly with plasminogen and urokinase and may mediate binding of these fibrinolytic components to cells.. Biochemistry.

[OCR_01438] Miles L. A., Dahlberg C. M., Plescia J., Felez J., Kato K., Plow E. F. (1991). Role of cell-surface lysines in plasminogen binding to cells: identification of alpha-enolase as a candidate plasminogen receptor.. Biochemistry.

[OCR_01443] Milligan G., Parenti M., Magee A. I. (1995). The dynamic role of palmitoylation in signal transduction.. Trends Biochem Sci.

[OCR_01447] Møller L. B. (1993). Structure and function of the urokinase receptor.. Blood Coagul Fibrinolysis.

[OCR_01451] Nakajima K., Hamanoue M., Takemoto N., Hattori T., Kato K., Kohsaka S. (1994). Plasminogen binds specifically to alpha-enolase on rat neuronal plasma membrane.. J Neurochem.

[OCR_01456] Nielsen B. S., Sehested M., Timshel S., Pyke C., Danø K. (1996). Messenger RNA for urokinase plasminogen activator is expressed in myofibroblasts adjacent to cancer cells in human breast cancer.. Lab Invest.

[OCR_01461] Parkkinen J., Rauvala H. (1991). Interactions of plasminogen and tissue plasminogen activator (t-PA) with amphoterin. Enhancement of t-PA-catalyzed plasminogen activation by amphoterin.. J Biol Chem.

[OCR_01466] Plow E. F., Miles L. A. (1990). Plasminogen receptors in the mediation of pericellular proteolysis.. Cell Differ Dev.

[OCR_01470] Rana A. P., Majumder G. C. (1987). Factors influencing the yield and purity of goat sperm plasma membranes isolated by means of an aqueous two-phase polymer system.. Prep Biochem.

[OCR_01475] Singleton T. P., Strickler J. G. (1992). Clinical and pathologic significance of the c-erbB-2 (HER-2/neu) oncogene.. Pathol Annu.

[OCR_01479] Stack M. S., Moser T. L., Pizzo S. V. (1992). Binding of human plasminogen to basement-membrane (type IV) collagen.. Biochem J.

[OCR_01483] Stonelake P. S., Jones C. E., Neoptolemos J. P., Baker P. R. (1997). Proteinase inhibitors reduce basement membrane degradation by human breast cancer cell lines.. Br J Cancer.

[OCR_01488] Thompson E. W., Paik S., Brünner N., Sommers C. L., Zugmaier G., Clarke R., Shima T. B., Torri J., Donahue S., Lippman M. E. (1992). Association of increased basement membrane invasiveness with absence of estrogen receptor and expression of vimentin in human breast cancer cell lines.. J Cell Physiol.

